# The Contribution of Adipose Tissue-Derived Mesenchymal Stem Cells and Platelet-Rich Plasma to the Treatment of Chronic Equine Laminitis: A Proof of Concept

**DOI:** 10.3390/ijms18102122

**Published:** 2017-10-11

**Authors:** Mario Angelone, Virna Conti, Cristiano Biacca, Beatrice Battaglia, Laura Pecorari, Francesco Piana, Giacomo Gnudi, Fabio Leonardi, Roberto Ramoni, Giuseppina Basini, Silvia Dotti, Sabrina Renzi, Maura Ferrari, Stefano Grolli

**Affiliations:** 1 Dipartimento Scienze Mediche Veterinarie, Università di Parma, Via del taglio, 10, 43126 Parma, Italy; marioangelonevet@libero.it (M.A.); virna.conti@unipr.it (V.C.); giacomo.gnudi@unipr.it (G.G.); fabio.leonardi@unipr.it (F.L.); roberto.ramoni@unipr.it (R.R.); giuseppina.basini@unipr.it (G.B.); 2 Veterinary Practitioner, Dipartimento Scienze Mediche Veterinarie, Università di Parma, Via del taglio, 10, 43126 Parma, Italy; cristiano.biacca@libero.it (C.B.); beatricechiarabattaglia@gmail.com.it (B.B.); laurapecorari.dvm@gmail.com (L.P.); f.piana.vet@gmail.com (F.P.); 3 Istituto Zooprofilattico Sperimentale della Lombardia e dell’Emilia Romagna, Via Bianchi 9, 25124 Brescia, Italy; silvia.dotti@izsler.it (S.D.); sabrina.renzi@izsler.it (S.R.); maura1.ferrari@libero.it (M.F.)

**Keywords:** mesenchymal stem cells, platelet-rich plasma, regenerative medicine, equine, laminitis

## Abstract

Laminitis, a highly debilitating disease of the foot in ungulates, is characterized by pathological changes of the complex lamellar structures that maintain the appendicular skeleton within the hoof. Laminitis is a multifactorial disease that involves perturbation of the vascular, hematological, and inflammatory homeostasis of the foot. Interestingly, the pathogenesis of the disease resembles what is observed in metabolic syndromes and sepsis-induced organ failure in humans and animals. We hypothesized that local administration of mesenchymal stem cells (MSCs) and platelet-rich plasma (PRP) might contribute to establishing an anti-inflammatory and pro-angiogenic environment, and could stimulate the injured tissue in order to restore its functional integrity. According to this assumption, an experimental protocol based on the local intravenous administration of adipose tissue-derived MSCs (aMSCs) in combination with PRP was developed for the treatment of horses affected by chronic laminitis. Nine horses with severely compromised venograms (showing grade III and IV laminitis) that had been unsuccessfully treated with conventional therapies were enrolled. aMSCs and PRP (15 × 10^6^ cells resuspended in 15 mL of PRP) were injected into the lateral or medial digital vein three times, at one-month intervals. The first administration was performed with allogeneic aMSCs, while for the following administrations, autologous aMSCs were used. There was no adverse short-term reaction to the intravenous injection of aMSCs. In the long term, venograms outlined, in all subjects, a progressive amelioration of the vascularization of the foot. An improvement in the structure and function of the hoof was also observed. No adverse events were reported during the follow-up, and the horses returned to a comfortable quality of life. Although the number of animals enrolled in the study is limited, both clinical observations and venography demonstrated an enhancement in the condition of all horses, suggesting that the regenerative therapies in chronic laminitis could be useful, and are worthy of further investigation.

## 1. Introduction

Laminitis is a disease affecting the hooves of ungulates, particularly in equines. It is a strongly debilitating disease that can leave an animal useless, quite often leading to the euthanasia of the patient. In this disease, the hoof (the horny capsule of the horse’s foot), and the lamina (a specialized tissue in charge of the deposition of the hoof), are involved [1].

The foot of the horse is a complex structure that must sustain a huge amount of weight and force [2]. Inside the hoof capsule, in the distal part of the skeleton the third phalanx (coffin bone) is enveloped by a type of epithelial tissue, the corium, that is responsible for the deposition of the hoof (coronary corium) and for the attachment of the hoof to the skeleton (laminar corium). 

The laminar corium originates from the outer surface of the coffin bone and produces the leaf-like lamellae (lamina), which suspends the coffin bone inside the hoof capsule by growing outward and merging with the inner structures of the hoof wall in a velcro-like fashion, so that the third phalanx and hoof are fixed together. Lamellar structures show an epidermis-like structure, a derma, and a very rich vascular bed with veins, arteries, and diffused capillarization and innervation. Laminitis is the inflammation of the lamina [1]. Edema, vascular compression, hypoxia and cellular necrosis as a consequence of inflammation can lead to laminar failure, thus compromising the fastening system between the hoof capsule and the third phalanx [1,3].

A great variety of conditions can lead to the onset of the disease. Laminitis can be a consequence of a primary disease, such as abdominal surgery, gastrointestinal disease, and pneumonia or any other disease causing sepsis. Moreover, it has been observed that it can also follow non-septic endotoxemia [4,5]. The term “systemic inflammatory response syndrome” is sometimes used to describe a condition where an animal has a systemic reaction, causing laminitis, in response to a local inflammation [4]. Furthermore, metabolic syndromes like insulin resistance and corticosteroid dysmetabolism can lead to laminitis. Finally, a frequent form of laminitis is associated with excessive carbohydrate intake in horses at free pasture [1,4,5].

Acute forms of laminitis cause lameness and pain; the horse is reluctant to move or does not move at all. No clear changes can be seen in radiographs. In chronic laminitis, the components of the lamina are detached: the chronic form is characterized by clear damage to the basement membrane of the derma so that the structure collapses. In this form, possible events are the rotation of the third phalanx, or sometimes sinking, when the phalanx, which is no longer fixed to the hoof wall, moves downward causing serious damage to the sole [6,7].

Both the acute and chronic form can have a poor prognosis, with a strong social and economic impact in the equine industry [6,8].

Whatever the triggering factors leading to the disease are, molecular and cellular events that play a key role in the pathogenesis of both acute and chronic laminitis are the release of cytokines and pro-inflammatory mediators, the induction and activation of metalloproteinases, and the degradation of the basement membrane [8,9,10,11]. In the course of laminitis, ischemic damage of the laminar tissues progressively compromises normal foot vascularization. Furthermore, hypoxia–reperfusion injury, endothelial instability, and tissue edema have been observed, leading to basement membrane deterioration and vascular compression and disruption [9,10]. The chronic phase of laminitis is characterized by an inadequate regenerative response, with laminar inflammation, neutrophil infiltration, and stromal cell activation [3,11].

To date, knowledge on the pathogenic mechanisms involved in the onset and progression of laminar failure is largely incomplete, with obvious consequences to both prevention and the therapies that can be applied [1,4,9].

Acute laminitis, frequently associated with a variety of primary diseases, is often the local result of a systemic inflammatory syndrome where metabolic dysfunction, endothelial and vascular alteration, and basement membrane degradation leads to laminar impairment or disruption. Vascular weakening is observed in both the acute and chronic phase, when endothelial cell swelling, erythrocyte accumulation, and leukocyte extravasation and infiltration are accompanied by edema and production of proinflammatory cytokines and bioactive molecules [1,11,12]. A local increase in Interleukin-1b (*IL-1b*), Interleukin-8 (*IL-8*), interleukin-6 (*IL-6*), and cyclooxygenase-2 (*COX-2*) mRNA levels has also been observed, while anti-inflammatory interleukin 10 (*IL-10*) gene transcription is downregulated [1,9]. Interestingly, Matrix-Metalloproteinases-2 (*MMP-2*) and Matrix Metalloproteinase-9 (*MMP-9*) genes, which are responsible for extracellular matrix degradation and hence basement membrane impairment, are upregulated [13]. Furthermore, an increase of Tumor Necrosis Factor α (*TNFα)*, Interleukin-6 (*IL-6*), Interleukin-8 (*IL-8*), and Interleukin-1 *β* (*IL-1 β)* gene expression has been observed in the lung and liver, pointing out a systemic increase in the inflammatory status of the animal [14]. Inflammatory mediators may activate processes known to damage the lamellar interface, by producing, for example, MMP-9 and MMP-2 [14,15]. Remarkably, it has been proposed that the molecular and cellular pathways that lead to laminar failure are similar to those observed in septic and/or toxic organ failure in human and animal models (i.e., rats) [16,17]. 

Mesenchymal stem cells (MSCs), a population of the stromal environment of a variety of mesenchyme-derived tissues, have been recently proposed as a therapeutic agent able to control several steps of organ failure, protecting tissues and organs from inflammatory damage. MSCs have demonstrated protective effects against tissue insults in acute lung injury models, acute respiratory distress syndrome, acute liver and renal failure, and acute brain injuries [18,19]. MSCs exhibit immunomodulatory and anti-inflammatory properties, exert anti-oxidative effects protecting against hypoxia /reperfusion injury, preserve the endothelial integrity, and promote angiogenesis [20,21,22]. Although the molecular pathways are not clearly defined, it has been demonstrated, both in vitro and in animal models, that MSCs can produce and secrete bioactive molecules, including interleukin-1 receptor antagonists (IL1-Ra, IRAPs), stromal cell-derived factor-1 (SDF-1), tumor necrosis factor-inducible gene 6 (TSG-6), and stanniocalcin-1 (STC-1), that are involved in these protective actions [18,19].

The aim of this work was to evaluate, as a proof-of-principle, a possible role of adipose tissue-derived MSCs (aMSCs) in the therapy of spontaneous clinical cases of equine laminitis. A restricted group of nine animals, previously treated with an unsatisfactory outcome, were enrolled in the study and treated by intravenous administration into the digital vein with aMSCs resuspended in autologous platelet-rich plasma (PRP), a blood-derived biomaterial whose activity in regenerative medicine has been demonstrated both in vitro models and in clinical applications [23]. The work describes the clinical procedure and the clinical outcome after 6 to 24 months for the different cases. Furthermore, gene expression analysis of proteins whose role in controlling inflammation-induced damage has been proposed, has been evaluated in equine aMSCs. The work is a first report suggesting that MSCs and PRP may be functional in the therapy of equine laminitis, probably by contributing to the stabilization of the vasculature and controlling the pro-inflammatory environment of the laminitic foot.

## 2. Results

### 2.1. Phenotypic Characterization of Adipose Tissue-Derived MSCs

A set of markers has been evaluated to describe, by means of semi-quantitative RT-PCR, the phenotype of aMSCs used in the present study. In particular, aMSCs expressed *CD90*, *CD105*, *CD44*, and *CD73*, while failing to express *CD45*, *CD31* and *CD34* (Table 1). The pattern of gene expression, although not quantitative, gave comparable results for each analyzed population.

In addition, we have also evaluated the expression of genes involved in the biological response of MSCs to tissue and organ injuries. With regard to this, aMSCs expressed *IL1-Ra*, *STC-1*, *TGS-6* and *SDF-1*, but failed to express SDF-1 receptor *CXCR4* (Table 1).

### 2.2. Quality Controls

None of the aMSCs batches used for the therapy was contaminated by bacteria, mycoplasma, viruses, or yeasts.

### 2.3. Safety of Clinical Procedures

The clinical status of each patient enrolled in the study was accurately monitored for the two weeks following each cell treatment, by evaluating the presence of edema within the distal portion of the limb where cells were injected, and eventual swelling, pain, or other symptoms not related to the laminitis. Temperature, breath and heart rate were evaluated twice a day. No local or systemic adverse reactions were observed after allogeneic (first administration) or autologous (second and third administration) aMSC injections. None of the horses showed clinical signs attributable to intravascular microcoagulation or thrombosis, a potential risk after intravenous injection of a high dose of aMSCs. In particular, in no case was a local reaction associated with acute vascular problems (hyperemia, temperature increase, edema, pain after palpation) observed. Furthermore, no abnormal findings attributable to the therapy were reported on the long term, i.e., over a period between 6 and 24 months, for any of the treated animals.

### 2.4. Efficacy and Outcome of the Therapy

All the horses enrolled in the study, at the moment of the hospitalization that preceded the treatment, had clinical status and diagnostic reports entailing a poor prognosis and a great risk of animal euthanasia. The use of the regenerative therapy, associated with a proper biomechanical approach, determined a satisfactory recovery of the functionality of the affected feet so that each animal could be dismissed in good general condition (see Supplementary Materials, files “BeforeTreatment.wmv” and “AfterTreatment.wmv”, illustrating Case 2 gait before and after treatment). Table 2 reports a synthetic description of each case.

All the animals enrolled in the study returned to their activity at six months from the first treatment. Seven animals out of nine were still performing their activity after one year. With regard to the long-term outcome, two animals had a recurrence of the laminitis and were euthanized, while three horses deceased as a result of causes unrelated to laminitis. These horses did not show symptoms related to laminitis. For Cases 8 and 9, the outcome >1 year is not yet available.

The evolution of the disease was monitored by means of clinical examination, radiography, and venography. Each patient showed a slight and constant increase in both movement and usage of the affected leg, and an improvement in the general physical condition. Typical signs of laminitis, such as bounding digital pulse, reluctance to move and walk, and impaired quadrupedal standing, as well as lameness and antalgic posture, gradually improved starting from the first aMSC/PRP injection. 

At the end of the therapeutic protocol, the macroscopical appearance of the laminitic hoof improved with regard both to the shape of the foot and hoof firmness. The hoof showed a significant growth rate during the therapy. 

Both the outer aspect at visual inspection and deep palpation of the laminitic foot highlighted a significant improvement within one month of the end of the therapeutic protocol.

**Figure 1:**
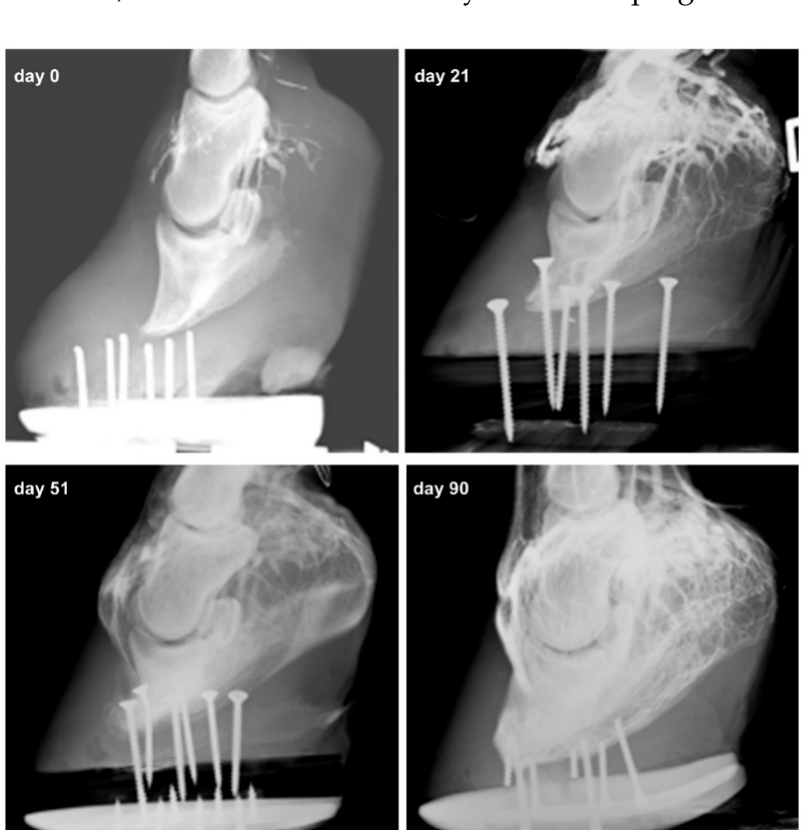
Case 2: Venograms of the right foot affected by laminitis at different time points. At 21 days after the first aMSC/PRP treatment, an increase in venous filling is observed. The venographic appearance of the digital vascular bed showed restored vascularization of the foot 90 days after the first aMSC/PRP treatment.

**Figure 2:**
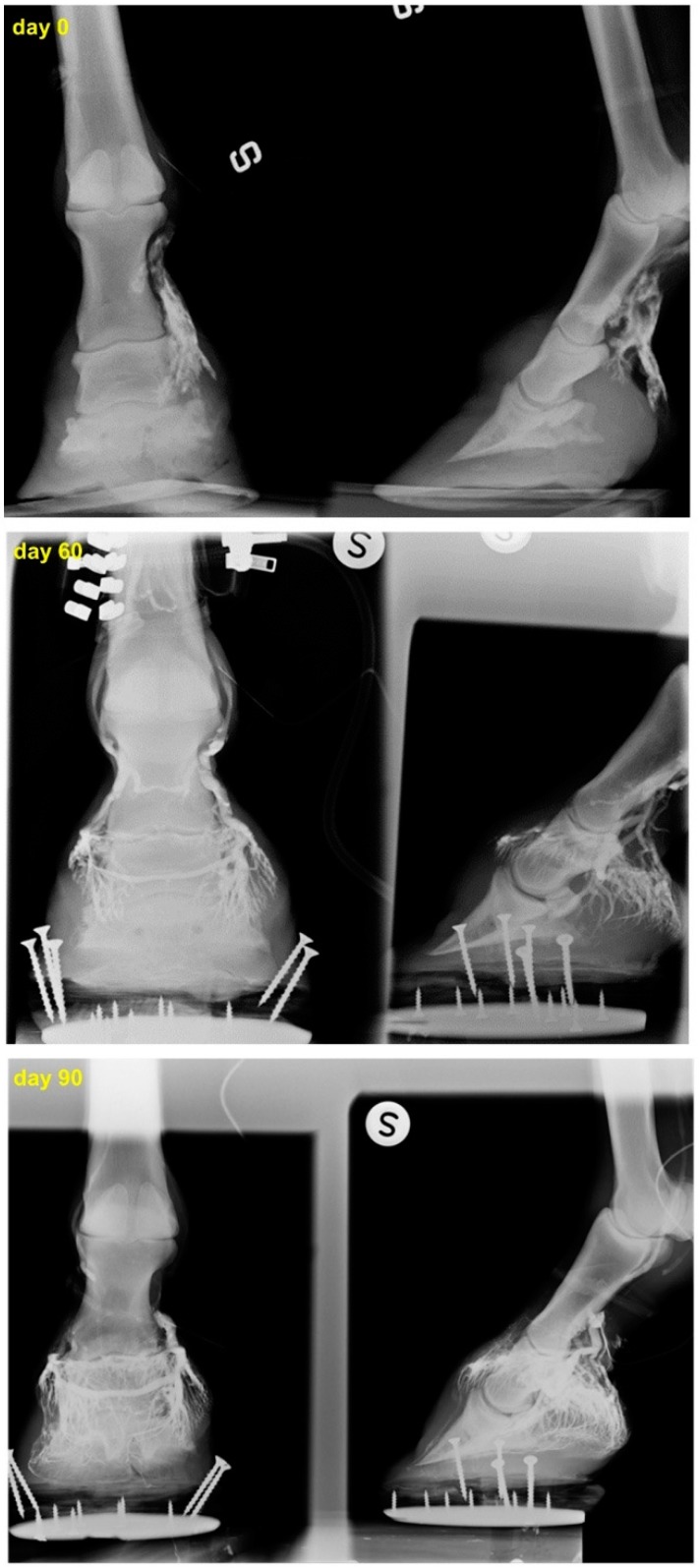
Case 8: venograms of the left foot. Increased venous filling is observed 60 days after the first aMSC/PRP administration. The venographic appearance showed a clear restoration of the vascular net 90 days after the first aMSC treatment.

Venographies showed that vascularization of the dorsal-distal portion of the finger, severely impaired in all the patients, was significantly ameliorated after the first two aMSC treatments, achieving almost complete restoration following the third treatment (Figure 1 and Figure 2). In particular, the venographies demonstrated, for each case, the progressive improvement of vascularization of the foot, made evident by the amelioration of the vascular net that, starting from the superficial and proximal region of the hoof, gradually involved the deep and distal layers, in some cases leading to the restoration of the foot´s vascular net up to the sole. The degree of vascular recovery, while diverse for the different clinical cases, has been characterized by a constant progression over time.

## 3. Discussion

The RT-PCR analysis of the aMSC phenotype substantially recapitulates what has already been described for horses, as well as for aMSCs from other species [24,25]. We have evaluated a panel of several marker genes comprising *CD45*, *CD29*, *CD44*, *CD73*, *CD105*, *CD34*, and *CD31*. Equine aMSCs express *CD90*, *CD44*, *CD105*, and *CD73*, and do not express *CD45*, *CD34* and *CD31*. Furthermore, we have evaluated the expression of genes involved in the protection of tissues against the damage induced by inflammation and hypoxia/reperfusion injury, i.e., *STC-1*, *TSG-6*, and *IL1-Ra* [18,19], or that have a role in the recruitment and homing of MSCs (*SDF-1* and its receptor *CXCR4*) [26], whose expression has not been previously reported for equine aMSCs cells. In particular, STC-1 exerts a tissue-protective role against tissue damage, blocking negative effects induced by reactive oxygen species (ROS) produced as a result of tissue hypoxia/reperfusion [27,28]. IL1-Ra is a receptor antagonist of the pro-inflammatory cytokine IL-1, thus having a specific protective role against inflammatory pathways. Secretion of IL1-Ra by MSCs has been demonstrated to reduce lung injuries induced by respiratory syndrome [18,29]. TSG-6 has anti-inflammatory action and, furthermore, a specific inhibitory function against MMPs [30]. Finally, SDF-1 and its receptor are key players in the recruitment of MSCs and endothelial cells in inflamed, as well as in damaged tissues [31,32]. Equine aMSCs express STC-1, IL1-Ra, TSG-6, and SDF-1. In contrast, equine aMSCs apparently do not express CXCR4, the receptor for SDF-1, in our culture condition. A loss of expression of the *CXCR4* gene has been reported for cultured MSCs in other species also, presumably as a consequence of in vitro culture conditions [33]. As a whole, these data suggest a potential tissue protective role of equine aMSCs, that in the case of chronic laminitis could contribute to the control of both local inflammation and impairment of laminar tissue.

During chronic laminitis, laminar tissue detachment causes an alteration of foot anatomy. As a result, vasculature, already compromised by inflammation, edema, and increased pressure, is even more endangered by the anatomical derangement of the foot. Vasculature deficit, therefore, could be a consequence of both anatomical damage and soft tissue sufferance and inflammation. We reasoned that MSCs could contribute to the therapy of laminitis by controlling inflammation, lowering ROS negative biological activity and MMP extracellular matrix degradation, and recruiting both local and circulating stem cells to restore tissue homeostasis. Furthermore, this assumption was in agreement with a recent work by Pascucci et al. [34] reporting that equine MSCs promote angiogenesis in vitro by means of membrane vesicle secretion, thus supporting the potential role of aMSC as a therapeutic for equine laminitis where venous and/or arterial vascularization is compromised. 

PRP was used in combination with aMSCs for the administration of the cells. PRP is considered a natural mixture of growth factors that produces clinical benefits when applied in wound healing, osteoarthritis, bone healing, and in regenerative plastic surgery [35]. The combined use of aMSCs and PRP has been proposed on the assumption that bioactive factors stored in the different platelet granules can contribute to, and reinforce, aMSC biological functions [23]. With regard to this point, it must be considered that since aMSCs and PRP have been administered in combination, their single contribution to the clinical amelioration of the laminitic foot could not be determined in the present investigations. From this point of view, the combination therapy could be considered a limitation of the study, as already pointed out in the literature [36,37,38]. Nonetheless, it must be underlined that the combination of the two biological therapeutics has been frequently adopted in recent years to develop regenerative medicine therapeutic protocols [35], and we opted for their contemporary intravenous administration with the aim of enhancing tissue healing and clinical outcome in severely affected animals which failed to respond to conventional treatments.

During the present study, a total of 42 intravenous injections of aMSCs resuspended in PRP were performed: four patients suffered monolateral laminitis and were injected three times, while five horses, affected by bilateral laminitis, were treated six times (see Table 3). 

The administration of aMSCs through the distal digital vein did not induce clinical signs related to adverse reactions. For each horse, the first administration was performed using allogeneic cells, to avoid the treatment delay of 3–4 weeks necessary to isolate and prepare a suitable number of autologous cells. The use of allogenic cells raises two potential risks: the transmission of viral and or/bacterial diseases, and the potential immune response against foreign cells. The safety of allogenic cells was evaluated by microbiological tests, excluding the presence of infectious agents as previously reported [39]. With regard to immune response, equine MSCs derived from adipose tissue as well as bone marrow tissue show immunosuppressive and immune-modulating features, in vitro [40]. Recently Kol et al. [41] reported that equine allogeneic MSCs do not induce clinical signs when administered intravenously, while Ardanaz et al. [42] demonstrated that repeated intra-articular injections of pooled allogeneic MSCs do not raise any clinical and inflammatory response. Recently Owens et al. [43] and Pezzanite et al. [44] demonstrated that allogeneic MSCs treatment induces alloantibody production in the horse, although this finding has not been associated to any adverse reaction in the short or long term outcome. As a whole, these data suggest the feasibility of the use of a single administration of allogeneic MSCs in the horse, although a better understanding of the immune response against allogeneic MSCs is mandatory. The present work also indicates that the repeated intravenous administration of aMSCs (each animal was injected at least three times) does not induce local undesired effects. In addition, the capillary thrombosis that might occur as a consequence of the injections [45] did not compromise vascular bed regeneration, as demonstrated by venography that indeed outlined an amelioration of the vascularization of the foot.

All the horses enrolled in the therapy had an improvement in the anatomical and physiological conditions of the laminitic foot. The quality and the shape of the hoof improved, as well as the animal’s mobility. Most of the animals showed a serious impairment of their capacity to stand in a normal stance or walk before the therapy. The treatment improved the welfare of the horses, giving each animal the chance to have a good quality of life. One of the horses, a mare, give birth to a healthy foal after therapy. According to these observations, the clinical improvement of the nine patients supports the hypothesis that aMSCs combined with PRP can be useful for ameliorating tissue function and homeostasis in the chronic laminitic foot. 

The pathogenic mechanisms underlying horse laminitis, as well as the tissue background or the systemic conditions of the animal that cause a predisposition to the onset of the disease, are still matter of debate [1,4]. A unifying model, explaining the different forms of the disease as well as their evolution, has not been proposed yet. Most probably, different pathogenic pathways converge in the onset of a pathological scenario where some key points, at the cellular or tissue level, justify the onset of the disease. Whole body (systemic) or organ specific toxicity, septic or not septic, can induce hoof lamina inflammation. White blood cell extravasation, MMP activation with degradation of the extracellular matrix components, edema, and vascular impairment gradually undermine lamina attachment, causing lamellar lengthening and rearrangement of dermal anatomy, leading to the loss of function of the foot. 

From this point of view, the assessment of the vascular changes associated to laminitis by means of venography is considered informative for evaluating both the stage and disease prognosis during and after the treatment [6,46]. This technique, in fact, allows the detailed visualization of the entire vasculature bed (veins, capillaries, arterio-venous anastomoses, and arterial circulation) of the foot [46]. In chronic laminitis, laminar tissue detachment causes alteration of foot anatomy and displacement of the third phalanx inside the hoof capsule (rotation and/or sinking). As a result, the vasculature, already compromised by inflammation and consequent edema, is even more endangered by this anatomical derangement. As a matter of fact, the recovery of foot vascularization that was observed by the venographic examinations can be considered both a cause and a sign of amelioration of foot functionality.

We propose that both aMSCs and PRP could have the potential to contribute to restore a functional vascular network by controlling inflammation, recruiting local cells to restore tissue, lowering ROS and MMP damage, stabilizing vascular wells, and promoting neoangiogenesis. In particular, MSCs have the potentiality to modulate these events both through their secretory ability and by the release of microvesicles. 

Restoring the functionality of the foot vasculature and dermal structures means improving the deposition of the hoof horn, that in turn reinforces soft tissue recovery. From this point of view, a correct shoeing of the laminitic foot is essential and should be considered an indispensable reinforcement of cellular therapy.

We are aware that the clinical results of our study are related to a low number of animals and that the study does not report controls. As a matter of fact, the recruitment of controls for the treatment of clinical cases of serious chronic laminitis (grade III–IV) like that proposed in the present investigation is ethically problematic. All the horses enrolled in the study had already been treated with conventional therapy (shoeing, etc.), and the owners expressed an explicit intention to recover the animals at least to an acceptable welfare standard. For each animal, the aim has been successfully achieved. From this point of view, results suggest that the use of a cell therapy for the treatment of equine chronic laminitis could be further investigated, both to set up more precise protocols and clinical procedures, and to elucidate the molecular and cellular events involved in the regenerative process.

## 4. Materials and Methods

### 4.1. Stem Cell Isolation from Adipose Tissue

Allogeneic cell preparation: cell isolation from adipose tissue and in vitro expansion was performed as previously described by Riccò et al. [36]. Briefly, adipose tissue samples (approximately 10 grams) for allogeneic cell preparation were collected from the intra-abdominal fat deposit of adult horses at the abattoir immediately after slaughtering. Two grams of tissue, minced under sterile conditions and washed several times with phosphate buffer saline solution (PBS) containing penicillin (1,000 IU/mL) and streptomycin (100 mg/mL) (Sigma-Aldrich Corp., ST. Louis, MO, USA) were then digested with 0.075% type I collagenase (Sigma-Aldrich) in Dulbecco’s modified Eagle’s low glucose medium (DMEM) (Sigma-Aldrich), under mild shaking, for 1 hour at 37 °C. A ratio of 5 mL of digestion medium for 1 gram of tissue sample was used. After digestion, tissue fragments were removed and cells suspension was filtered through a 50-μm nylon mesh and then centrifuged at 250× *g* for 10 min. The supernatant was removed and the cell pellet resuspended in DMEM containing 10% *v*/*v* fetal bovine serum (FBS) (Sigma-Aldrich), 1000 IU/mL penicillin (Sigma-Aldrich), and 100 mg/mL streptomycin (Sigma-Aldrich). Finally, cells were seeded in 25 cm^2^ tissue culture flasks at the concentration of 5.0 × 10^4^ cells/cm^2^, and incubated at 37 °C in 5% CO_2_. After 48 h, non-adherent cells were removed by replacing the culture medium. Medium was renewed every 72 h thereafter. After the first passage (days 7–10), subsequent subcultures were performed when cells reached about 80% confluence. A maximum of four serial passages was performed. Cells were stored in liquid nitrogen at 4 × 10^6^ cells/mL freezing medium.

Autologous cells preparation: Adipose tissue samples were collected in accordance with local animal protection guidelines. Surgical procedures were performed in standing conscious horses with appropriate sedation and local analgesia. Detomidine (2–5 µg/kg; Domosedan, Orion Corporation, Espoo, Finland) combined with butorphanol (0.01 mg/kg; Nargesic, ACME, Reggio Emilia, Italy) was given intravenously. Lidocaine (1 mg/kg; Lidocaina 2%, ATI, Bologna, Italy) was injected subcutaneously in the ventral region of the tail base 15 min after detomidine/butorphanol injection. Approximately 2 g of adipose tissue were collected from the ventral region of the tail base, as described by Carvhalo et al. [47] and Barberini et al. [48]. Tissue samples were processed as previously described for allogeneic cell preparation.

### 4.2. Platelet-Rich Plasma (PRP) Preparation

PRP was obtained from autologous whole venous blood collected with adenine–citrate–dextrose–acid solution (ACD) anticoagulant (Terumo blood bag, Terumo Europe, Zaventem, Belgium). Fresh blood samples were aliquoted in 50-mL tubes and centrifuged at 150× *g* for 20 min at 4 °C. The erythrocyte fraction and the buffy coat were discarded, while the plasma, enriched with platelets, was further centrifuged at 800× *g* for 15 min. The platelet pellet was collected and platelets were then counted (CELL-DYN 3500R hematology analyzer, Abbott, Abbott Park, IL, USA) and diluted to a final concentration of 1.0 × 10^9^ platelets/mL.

### 4.3. aMSC Preparation for Administration

One or two hours before implantation, 15 × 10^6^ cells were thawed, washed with DMEM without serum, and then resuspended in 15 mL of autologous PRP. Cell viability was assayed by Trypan-blue (Sigma-Aldrich) staining.

### 4.4. Quality Controls

All cell batches were tested for microbial contamination, as previously reported [39]. Mycoplasma infection was evaluated using the commercial MycoSensor PCR Assay Kit (M-Medical S.r.l., Milan, Italy). Bacteria, fungi, and yeast contaminations were investigated following inoculation of 2 mL of each cell suspension in microbiological media (Sabouraud agar, triptic soy agar and/or brain heart infusion, incubated at 30 and 37 °C, respectively). In order to detect possible viral contamination, all cell samples were cocultured with susceptible cell lines. They were incubated at 37 °C in 5% CO_2_ for 5 days, in order to observe cytopathic effects. These cell lines are stored in the IZSLER Cell Culture Bank (Brescia, Italy). In particular, equine *Herpesvirus*, *Arterivirus*, and *Flavivirus* infections were detected following cell sample inoculation of the equine dermis (EDe, ) and rabbit kidney (RK13, Cod. BSCL74). Furthermore, *Flavivirus* and non-cytopathic BVD, after cultivation in EDe cells, were evaluated by Real-time PCR.

### 4.5. Selection and Description of Clinical Cases

Nine horses of different breeds, sex, age and activity, were enrolled in the study (Table 3). Informed consent was obtained from the owners of the animals. Procedures were approved by the Italian Ministry of Health (project name: “Therapeutic application of allogeneic mesenchymal stromal cells in veterinary medicine”, code: CSM AL; supervisor: Istituto Zooprofilattico della Lombardia ed Emilia Romagna, Brescia).

All the horses had been unsuccessfully treated with conventional therapies and enrolled in the study following an informed consent from the owner. Venography stage was determined following the classification proposed by Floyd [49]. 

The horses enrolled in the study were subjected to an orthopaedic examination which allowed the diagnosis of a severe form of laminitis. The prognosis of eight horses was poor prior to hospitalization. All patients had severe clinical symptomatology and were not responsive to traditional therapies.

Eight patients (cases 1, 2, 3, 5, 6, 7, 8, and 9) showed serious difficulties in walking and maintaining standing posture; they had bedsores in different regions of the body (particularly at the level of the bony protuberances) secondary to the prolonged decubitus; they had also generalized cachexia.

In cases 1, 3, 5, 6, 7, 8, and 9 there was a prolapse of the third phalange; this part of the finger was exposed as a consequence of the collapse of the corneal sole. These same horses spilled purulent material from the laminitic feet. In these patients, it was necessary to carry out specific therapeutic procedures (surgical debridement of necrotic area, loco-regional and systemic antibiotic treatment, reconstruction of the dorsal part of the hoof) to stabilize the severe alterations encountered.

All the subjects of the study received a specific and personalized biomechanical therapeutic approach. An unconventional method was used, applying materials which do not damage the hoof and avoiding mechanical trauma typical of classical techniques. This choice has been found to be useful both to minimizing animal pain and to reducing stress to already heavily compromised tissues.

Instrumental investigation: all horses were subjected to a complete radiographic examination of the foot that provides the four orthogonal projections. Venographies were also performed by injecting each foot with 20 mL of iomeprolo 300 mg/mL (Iomeron 300, Bracco Imaging Italia, Milano, Italy) from digital veins according to D’Arpe et al. [50]. Venograms were evaluated following the classification proposed by Floyd [49]. The venography stage for each animal at the referral is reported in Table 3.

### 4.6. aMSC Administration

Before cell administration, the horses were sedated by intravenous administration of detomidine chlorohydrate (2–5 µg/kg; Domosedan, Orion Corporation, Espoo, Finland) and butorfanol (0.01 mg/kg; Nargesic, ACME, Reggio Emilia, Italy). aMSCs were injected intravenously into the digital palmar vein in the standing horse. 

Briefly, after tricotomy and disinfection of the region, a tourniquet was applied just proximally from the metacarpophalangeal joint. aMSCs (15 × 10^6^) resuspended in 15 mL of PRP were injected under sterile conditions by means of a 21-gauge butterfly needle, into the lateral or medial digital vein. The tourniquet was maintained in situ for 20 min following injection. After tourniquet removal, a protective bandage was applied.

Clinical examination has been performed every four hours for three days following cell administration, and then every eight hours until patient discharge. Each animal was treated three times at one-month intervals: the first time with allogeneic aMSCs, and then twice with autologous cells. Cell administration was preceded (7 days) by venography to assess foot condition. 

### 4.7. Semi-Quantitative RT-PCR

The assessment of gene expression was performed by reverse-transcription-PCR (RT-PCR). Total RNA was extracted from 1.5 × 10^6^ cells at P3 (80% of confluence) using the Nucleospin^®^ RNA II kit (Macherey-Nagel, Duren, Germany) following the user manual. cDNA was prepared by retro-transcription of 1.5 µg total RNA using RevertAit™ First Strand cDNA Synthesis Kit (ThermoFisher Scientific, Waltham, MA, USA). Each PCR was performed using 2 µL of cDNA. The final PCR mixture contained 1× amplification buffer with 2.5 mM MgCl2, 0.2 mM of each dNTP (ThermoFisher Scientific), 0.25 µM primer forward and reverse, and 1 U Dream Taq (ThermoFisherScientific) in a final volume of 25 µL. Table 4 reports the list of genes that were analyzed, their accession number, the sequence of forward and reverse primers, and the length of the relative amplicons. The products of the RT-PCR were separated on agarose gel (1.5% *p*/*v*) in TAE buffer (Tris-acetate-EDTA buffer containing 40 mM Tris, 20 mM acetic acid, and 1 mM EDTA, pH 8.0) stained with 3.5 µL ethidium bromide (10 mg/mL) (Sigma-Aldrich). Amplicons were visualized using UV light with a trans-illuminator and photographed. The analysis was repeated with two different replicates for each tissue sample. A semi-quantitative analysis of the expression of genes was performed evaluating the optical density of each positive band by means of the ImageJ image processing software (https://imagej.nih.gov/ij/index.html) normalized to the expression of the housekeeping gene β-actin.

## 5. Conclusions

Although the low number of cases enrolled in this study prevents us from generalizing the results and establishing a clear cause/effect between aMSC/PRP therapy and foot amelioration, the work suggests that regenerative medicine can be further explored in the treatment of chronic laminitis, when conventional therapies are not adequate. As a matter of fact, the treatment allowed the patients to recover to the point of good general condition. The horses returned to a good quality of life and some of them returned to their previous level of activity. These results can be considered of clear importance, especially for animals not used for sport, where the main objective is good quality of life. A further aspect of the study is the suggestion that naturally-occurring laminitis can provide an interesting model for the study of the contribution of MSCs to tissue and organ homeostasis. The complex pattern of molecular and cellular events that leads to the derangement of the epithelial layer and the dermal lamellae of the horse foot has been compared to what is observed in human septic and/or traumatic organ failure. From this point of view, the treatment of laminitis by means of cell therapies can provide a spontaneous disease model for comparative/translational medicine and help to understand the clinical potential of MSCs and PRP in term of both safety and efficacy, with a view to their applications to human medicine.

## Figures and Tables

**Table 1:** Gene expression analysis of adipose tissue-derived mesenchymal stem cells (aMSCs) by means of semi-quantitative RT-PCR. Legend: − not expressed; + target gene expression <25% of β actin expression; ++ target gene expression 25–50% of β actin expression; +++ target gene expression >50% β actin expression. Results are the average of analysis of three different aMSC populations.

Marker Gene	Expression in Equine aMSCs	% Target Gene vs β actin Gene Expression
*CD44*	positive	+++
*CD29*	positive	+++
*CD90*	positive	+++
*CD45*	negative	−
*CD34*	negative	−
*CD73*	positive	++
*CD13*	positive	+++
*CD133*	positive	++
*CD105*	positive	++
*CD31*	negative	−
*Oct-4*	positive	+
*IL1-Ra*	positive	++
*SDF-1*	positive	++
*CXCR4*	−	−
*TSG-6*	positive	+++
*STC-1*	positive	++

**Table 2:** Long term outcome of therapy for laminitic horses following aMSC and platelet-rich plasma (PRP) treatment. Legend: F = female, G = gelding.

Patient	Breed	Age	Sex	Activity	6-Month Outcome	1-Year Outcome	>1 Year Outcome (1–3 Years)
CASE 1	QUARTER HORSE	18	F	MARE	returned to activity	still in activity	deceased (intestinal colic)
CASE 2	QUARTER HORSE	14	F	MARE	returned to activity	still in activity	deceased
CASE 3	THOROUGHBRED	13	G	PLEASURE	returned to activity	still in activity	still in activity
CASE 4	QUARTER HORSE	10	F	PLEASURE	returned to activity	still in activity	still in activity
CASE 5	WARMBLOOD	18	G	PLEASURE	returned to activity	still in activity	deceased (intestinal colic)
CASE 6	WARMBLOOD	15	F	PLEASURE	returned to activity	recurrence of laminitis	euthanized
CASE 7	DUTCH WARMBLOOD	21	F	PLEASURE	returned to activity	recurrence of laminitis	euthanized
CASE 8	THOROUGHBRED	15	G	PLEASURE	returned to activity	still in activity	not applicable
CASE 9	PONY	16	G	PLEASURE	returned to activity	still in activity	not applicable

**Table 3:** Description of clinical cases enrolled in the study. Legend: F = female; G = gelding.

Patient	Breed	Age	Sex	Activity	Laminitis Stage	Venography Stage	Prognosis (before Treatment)
CASE 1	QUARTER HORSE	18	F	MARE	BILATERAL ROTATION	4	POOR
CASE 2	QUARTER HORSE	14	F	MARE	BILATERAL CHRONIC ROTATION	4	POOR
CASE 3	THOROUGHBRED	13	G	PLEASURE	MONOLATERAL SINKING AND ROTATION	4	POOR
CASE 4	QUARTER HORSE	10	F	PLEASURE	MONOLATERAL SINKING	3	GUARDED PROGNOSIS
CASE 5	WARMBLOOD	18	G	PLEASURE	BILATERAL SINKING AND ROTATION	3	POOR
CASE 6	WARMBLOOD	15	F	PLEASURE	MONOLATERAL SINKING AND ROTATION	3	POOR
CASE 7	DUTCH WARMBLOOD	21	F	PLEASURE	BILATERAL SINKING AND ROTATION	3-4	POOR
CASE 8	THOROUGHBRED	15	G	PLEASURE	MONOLATERAL ROTATION	3	POOR
CASE 9	PONY	16	G	PLEASURE	BILATERAL SINKING AND ROTATION	4	POOR

**Table 4:** Gene name, accession number, primer sequences and amplicon size of genes used for expression analysis of aMSCs.

Marker	Accession Number	pRIMER SEQUENCE	AMPLICON SIZE
*β-ACTIN*	AF035774.1	Fw: ACCCCGTGCTGCTGACCGA Rv: GCAGAAGGAGATCACAGCCCT	658 bp
*CD44*	X66862.1	Fw: CAGACCTGCCCAACGCCTTCGAGGGAC Rv: CAGAGCCAGGGCCAGGAGGGACGCC	440 bp
*CD29*	NM_001301217.1	Fw: ACAGATGCCGGGTTTCACTTTGC Rv: CCATTTTCCCCTGTTCCCATTCACCC	405 bp
*CD90*	EU881920	Fw: ATCGCTCTCCTGCTGACAGT Rv: CGGAGTTCGCATGTGTAGAG	302 bp
*CD45*	XM_008543985.1	Fw: TCCATGCAGATATTTTGTTGGACAC Rv: ATTGATGGCCAGTATTCTGCACACTTG	409 bp
*CD34*	XM_005609830.2	Fw: ATGCTGGTCCGCAGGGGCGCGCGC Rv: GGGCAAGGAGCAAGGAGCACAC	684 bp
*CD73*	XM_001500115	Fw: CAAAAAGGCCAACTTTCCAA Rv: AACCTTCCGTCCATCATCAG	430 bp
*CD13*	NM_001150	Fw: GGCAGATGACCTGGCGGGC Rv: ACCACCCGCTCCTTGTTG	591 bp
*CD133*	XM_001498679	Fw: TGTGTGGGACTACCGTTTCA Rv: TAGGTGGTGATTTGCCACAA	512 bp
*CD105*	XM_001500078	Fw: GCTGACGACAGAGATGACCA Rv: TCCTGGGATACAGGGCTATG	484 bp
*CD31*	NM_00110165	Fw: AAAGGGCCCAATACATTT Rv: GCAGGTATAGTGCCCGCTGT	686 bp
*Oct-4*	XM_001490108.2	Fw: TCCCAGGACATCAAAGCTCAGA Rv: TCTGGGCTCTCCCATGCATTCAAAC	321 bp
*IL1-Ra*	XM_005599766.2	Fw: ATGGAAATCCGCAGGCGTTCTG Rv: CTACTGGTCCTCCTGGAGGTAG	531 bp
*SDF-1*	KF612401.1	Fw: ATGAACGCCAAGGTCGTCGCCG Rv: CTTGTTTAAAGCTTTCTCCAGGTAC	359 bp
*CXCR4*	XM_001490165	Fw: CTACACAGTCAACCTCTCCAGC Rv: CTGCTCACAGAGGTGAGTGCATGC	597 bp
*TSG-6*	NM_001081906.1	Fw: ATGATCATCTTAATTTACGTACTTG Rv: TTATAAATGGGAAAACTTGG	845 bp
*STC-1*	XM_001493195.4	Fw: TGATCAGTGCTTCTGCAACC Rv: TCACAGTCCAGTAGGCTTCG	466 bp

## Supplementary materials

Supplementary materials can be found at www.mdpi.com/1422-0067/18/10/2122/s1. Two short videos of Case 2 recorded before (file name: beforetreatment.wmv) and after (file name: aftertreatment.wmv) therapy are uploaded as supplementary material. The files describe the general physical condition of the patient and its ability to walk.
